# Association Between Obesity and Serum Leptin Levels in Brazilian Female Shift Workers

**DOI:** 10.3390/diseases13120401

**Published:** 2025-12-15

**Authors:** Raquel Toresan Andretta, Janaína Cristina da Silva, Anderson Garcez, Ingrid Stähler Kohl, Karina Giane Mendes, Thais Basso, Maria Teresa Anselmo Olinto, Heloísa Theodoro

**Affiliations:** 1 Post-Graduate Program in Health Sciences, University of Caxias do Sul (UCS), Caxias do Sul 95070-560, RS, Brazil; rtandretta@ucs.br (R.T.A.); kgmendes@ucs.br (K.G.M.); nutricionistathaisb@gmail.com (T.B.); 2 Post-Graduate Program in Collective Health, University of Vale do Rio dos Sinos (Unisinos), São Leopoldo 93022-750, RS, Brazil; jcsilvanutri2@gmail.com; 3 Post-Graduate Program in Medical Sciences: Endocrinology, Federal University of Rio Grande do Sul (UFRGS), Porto Alegre 90610-264, RS, Brazil; adsgarcez@gmail.com (A.G.); ingridkohl.nutri@gmail.com (I.S.K.); 4 Post-Graduate Program in Food, Nutrition and Health, Federal University of Rio Grande do Sul (UFRGS), Porto Alegre 90610-264, RS, Brazil

**Keywords:** leptin, obesity, shift work, women’s health

## Abstract

Background: Leptin is a hormone that plays a crucial role in regulating energy homeostasis and it is associated with adiposity. Women engaged in work shifts are often exposed to circadian disruption and metabolic changes that may contribute to increased adiposity and hormonal imbalance. Thus, this study aimed to investigate the association between general and abdominal obesity and serum leptin levels among female shift workers. Methods: This cross-sectional study included a sample of 302 female employees from a group of plastic manufacturing industries in southern Brazil. Serum leptin levels were measured, with values > 15.2 ng/mL classified as elevated. General obesity (body mass index ≥ 30 kg/m^2^) and abdominal obesity (waist circumference ≥ 88 cm) were assessed. Associations were examined using Poisson regression with robust variance. Results: The mean age of participants was 35.4 ± 10.1 years. The mean serum leptin concentration in the sample was 33.6 ng/mL (95% Confidence Interval [CI]: 30.6–36.6), and the prevalence of altered serum leptin levels was 78.1% (95% CI: 73.5–82.8). After adjustment for potential confounders, women with obesity showed a 63% higher probability of having elevated leptin levels (Prevalence Ratio [PR] = 1.63; 95% CI: 1.32–2.02; *p* < 0.001) compared with those without obesity. Additionally, significant associations were observed with work shift and physical activity. However, abdominal obesity was not statistically significant after adjustment. Conclusions: Obesity was independently associated with elevated serum leptin levels among female shift workers, suggesting that excess adiposity remains a key determinant of leptin dysregulation in this population.

## 1. Introduction

Leptin is a hormone that plays a crucial role in regulating energy homeostasis and is primarily synthesized in white adipose tissue [1,2]. Circulating leptin levels are positively associated with adiposity [3]. Individuals with obesity often exhibit elevated levels of free, biologically active leptin in the brain, which may increase the likelihood of leptin resistance [4]. Leptin concentrations in the cerebrospinal fluid are associated with both plasma leptin levels and higher body mass index (BMI) [4]. Additionally, evidence indicates that the soluble leptin receptor is regulated by sex, adiposity, hormonal status, and recombinant human leptin (rhLeptin) administration [5], a regulation that has important implications for leptin’s biological activity, as the soluble leptin receptor is the principal leptin-binding protein and a key determinant of biologically active free leptin [5]. 

The intraday dynamics of circulating serum leptin levels follow a robust circadian pattern, characterized by low morning concentrations that progressively rise throughout the day and culminate in a pronounced nocturnal peak [6,7,8]. This rhythmic profile has been consistently documented across diverse metabolic phenotypes and is shaped by interactions among sleep–wake cycles, postprandial insulin signaling, feeding behavior, and broader mechanisms of energy homeostasis [6,7,8].

Leptin is a major regulator of food intake and energy balance through its action on leptin receptors (LEP-R) in the central nervous system [1,9,10]. Upon binding to its long-isoform receptor (LepRb) in specific hypothalamic neurons—particularly within the arcuate nucleus—leptin activates intracellular signaling cascades, most notably the JAK2–STAT3 pathway, which modulate neuropeptides that suppress appetite and increase energy expenditure [11,12,13]. More specifically, leptin signaling stimulates the expression of anorexigenic peptides such as α-melanocyte-stimulating hormone (α-MSH) while inhibiting orexigenic neuropeptides including neuropeptide Y (NPY) and agouti-related peptide (AgRP), thereby promoting satiety and reducing caloric intake [13,14,15]. 

Through this feedback mechanism, circulating leptin levels, which are proportional to adipose tissue mass, inform the brain about energy stores and adjust hunger and energy expenditure accordingly [1,9]. However, in individuals with obesity, leptin levels are often elevated, yet this does not induce satiety due to peripheral or central leptin resistance [16]. This resistance disrupts the balance between anorexigenic and orexigenic pathways, diminishing satiety and promoting weight gain [16,17]. Moreover, individuals with overweight or obesity tend to consume highly palatable foods, which enhance triglyceride synthesis and, consequently, leptin production by white adipose tissue [18].

The demand for shift work, particularly night shifts, has increased with technological advancements. To meet this demand, approximately 20 million people work night shifts, representing nearly 20% of the global workforce [19]. Night work has been linked to various adverse health outcomes, primarily resulting from circadian misalignment [20,21]. Factors such as nighttime eating habits, exposure to artificial light, and reduced physical activity may act as key contributors [22].

Circadian rhythm disruption is an important modulator of adipocyte-derived leptin secretion [23]. Disturbances in sleep–wake cycles, light exposure, or shift work can desynchronize central and peripheral clocks, altering leptin amplitude and rhythmicity [23]. Moreover, disruption of circadian rhythms due to shift work may promote the development of metabolic disorders, including elevated leptin levels [24], largely driven by the predominance of food intake during nighttime hours [25]. 

Given the above, this study aimed to investigate the association between general and abdominal obesity and serum leptin levels among female shift workers. Understanding this relationship is relevant, as women engaged in work shifts are often exposed to circadian disruption and metabolic changes that may contribute to increased adiposity and hormonal imbalance. In this context, this study provides a scientific investigation in a specific sample of shift-working women. We hypothesized that circulating leptin levels would be positively associated with adiposity in female shift workers.

## 2. Materials and Methods

### 2.1. Study Design and Population

This cross-sectional study was carried out with a sample of female employees working in an industrial complex dedicated to the production of plastic goods and household items, located in the metropolitan area of Porto Alegre, Rio Grande do Sul, Brazil. The investigation was embedded within a broader research initiative titled Health Conditions of Female Shift Workers: A Longitudinal Study on Occupational Health (ELO Saúde), which received prior approval from the Research Ethics Committee of the University of Vale do Rio dos Sinos (CAAE: 53762521.7.0000.5344; Approval No. 5.275.921). All participants provided written informed consent, and all ethical standards regarding confidentiality and anonymity were upheld in accordance with the principles of the Declaration of Helsinki.

### 2.2. Sample and Sampling

All female employees aged 18 years or older were deemed eligible for participation. The study encompassed workers from both production and administrative sectors of the company. Exclusion criteria included pregnancy at any gestational stage, temporary leave from work, and employment duration of less than three months. Among the 546 eligible workers, 452 women completed the interview process after accounting for refusals and losses. All participants were subsequently invited to provide blood samples, from which serum leptin levels were analyzed. Laboratory data were obtained for 302 women, comprising 232 production workers assigned to fixed six-day workweek shifts and 70 administrative workers. This sample size provided 80% power to detect prevalence ratio (PR) effect sizes of at least 0.14 with 95% confidence intervals (CI).

### 2.3. Data Collection and Instruments

Data collection took place between August 2022 and March 2023. A standardized, pre-coded, and pre-tested questionnaire was administered through in-person interviews conducted at the participants’ workplace. Anthropometric measurements were obtained immediately after each interview. All interviewers received formal training, and a pilot study was carried out to validate the instruments and ensure interviewer proficiency. To guarantee data reliability, approximately 10% of the interviews were reassessed via telephone using a brief version of the questionnaire containing stable-response items. Laboratory analyses were performed by a contracted certified company. Biological samples were collected in the morning (between 7:00 and 10:00 a.m.) by trained professionals in a designated area at the workplace or at the participants’ residences. Prior to collection, participants were instructed to fast for a minimum of 8 and a maximum of 12 h, avoid alcohol consumption for 72 h, refrain from caffeine intake, and abstain from vigorous physical activity for at least 24 h. All data were coded and verified by the research supervisors.

### 2.4. Outcome: Serum Leptin

Serum leptin concentrations were measured from serum samples with a minimum required volume of 0.5 mL. The samples remained stable for up to 30 days when stored frozen between −5 °C and −25 °C, preferably at −20 °C. Leptin quantification was performed using the Enzyme Immunoassay (EIA/ELISA) technique, and results were expressed in ng/mL. The assay followed the manufacturer’s protocol. Serum samples, standards, and controls were incubated in microplate wells pre-coated with anti-leptin antibodies, washed, and then exposed to an enzyme-conjugated secondary antibody. After addition of a chromogenic substrate, absorbance was measured using a microplate reader, and leptin concentrations were determined from a standard curve. Reference ranges for normal serum leptin levels were defined as 0.5–15.2 ng/mL, while values exceeding 15.2 ng/mL were classified as elevated [26].

### 2.5. Main Exposure: General and Abdominal Obesity

Anthropometric variables included nutritional status, evaluated using Body Mass Index (BMI), calculated as body weight in kilograms divided by height in meters squared. Weight and height were measured with a digital anthropometric scale (Omron^®^ model HN-289; capacity 150 kg; precision 100 g) and a portable stadiometer (Balmak^®^; capacity 2.1 m; precision 1 mm). All measurements were performed in duplicate, ensuring participants were in an upright position, barefoot, with arms alongside the body, and wearing light or minimal clothing. Based on BMI values, women were categorized as normal weight (BMI < 25 kg/m^2^), overweight (25 ≤ BMI < 30 kg/m^2^), or obesity (BMI ≥ 30 kg/m^2^) [27].

Abdominal obesity was evaluated using waist circumference (WC) measurements expressed in centimeters. WC was determined with a non-elastic measuring tape accurate to 1 mm, positioned directly on the skin at the midpoint between the lower margin of the last rib and the iliac crest. Participants were instructed to stand upright with a relaxed abdomen and arms resting alongside the body. Two measurements were obtained for each participant, and the mean value was used for analysis. Women were classified as having abdominal obesity when their waist circumference was greater than or equal to 88 cm (WC ≥ 88 cm) [28,29].

### 2.6. Covariates

Information on demographic, socioeconomic, behavioral, reproductive, health, and occupational characteristics was collected to describe the study population and control for potential confounding variables in the multivariable analysis. The demographic and socioeconomic variables considered included: age in completed years at the time of the interview and categorized into age groups (18–30, 31–40, ≥41 years); self-reported skin color categorized as White and Other (Black, Brown, Indigenous, and Yellow [Asian]); marital status (self-reported and classified as without a partner [single / separated / divorced / widowed] or with a partner [married / cohabiting]); and educational attainment reported in completed years of study (≤8 [Primary education], 9–11 [Secondary education], ≥12 [Technical/Higher education] years of study). Reproductive and health-related variables included: menstruation, assessed through self-reported menstruation in the past 12 months and classified as ‘no’ and ‘yes’; use of sleep medication—classified as ‘no’ (no reported use) and ‘yes’ (reported use of medications); use of medications for Diabetes Mellitus—classified as ‘no’ (no reported use) and ‘yes’ (reported use); and self-rated health, assessed using a five-point Likert scale and classified as: excellent/very good, good, and fair/poor. Shift work was the occupational characteristic evaluated in the present study. The production sector operated on three fixed shifts—morning (6:00 a.m.–2:00 p.m.), afternoon (2:00 p.m.–10:00 p.m.), and night (10:00 p.m.–6:00 a.m.)—while administrative employees worked daytime hours (7:00 a.m.–7:00 p.m.). Based on recorded clock-in and clock-out times, participants were classified as day-shift (6:00 a.m.–10:00 p.m.) or night-shift (10:00 p.m.–6:00 a.m.) workers. Behavioral characteristics investigated included: leisure-time physical activity, assessed through self-reported practice of any physical activity for leisure, sport, or exercise in the past week (excluding commuting), categorized as ‘no’ and ‘yes’; and number of daily meals, reported by the participant and categorized as ≤3 or ≥4 meals per day. 

### 2.7. Statistical Analyses

Data entry was conducted using EpiData software, version 3.1 (Centers for Disease Control and Prevention, Atlanta, GA, USA), employing a double-entry procedure followed by consistency checks to ensure data accuracy.

Descriptive statistics were applied to summarize the characteristics of the sample and the distribution of altered serum leptin levels. Numerical variables were expressed as means and standard deviations, whereas categorical variables were presented as absolute and relative frequencies. Pearson’s chi-square test was used to assess differences in the prevalence of altered serum leptin levels, while *t*-tests or one-way ANOVA were employed to compare mean serum leptin values across groups defined by sample characteristics.

For the multivariate analysis, Poisson regression with robust variance was applied following a hierarchical analytical framework [30], developed based on evidence from the literature. Variables with a *p*-value < 0.20 were included and retained at each level of the model. The analytical hierarchy was structured as follows: Level 1: Sociodemographic and reproductive variables; Level 2: Variables from Level 1 with *p* < 0.20 (menstruated in the last 12 months) plus work shift; Level 3: Variables from Level 2 with *p* < 0.20 (menstruated in the last 12 months and work shift) plus physical activity, number of meals, and self-rated health; Level 4: Variables from Level 3 with *p* < 0.20 (menstruated in the last 12 months, work shift, and physical activity) plus use of sleep medication, use of medication for diabetes mellitus, BMI classification, and abdominal obesity.

An additional analysis was performed to investigate the correlation between leptin levels and BMI. The non-normal distributions of both variables were evaluated and confirmed using the Shapiro–Wilk test (*p* < 0.001). Consequently, Spearman’s rank correlation coefficient (rho) was employed to assess the relationship between these non-normally distributed continuous variables.

All statistical analyses were performed using SPSS^®^ software, version 21.0 (IBM Corp., Armonk, NY, USA), with associations presenting a *p*-value ≤ 0.05 considered statistically significant.

## 3. Results

A total of 302 female workers aged 18 to 64 years (mean age: 35.4 ± 10.1 years) were included in the final analysis. Table 1 summarizes the general characteristics of the study population. Approximately 35.1% of the participants were aged 18–30 years, 69.5% self-identified as white skin color, and 52.0% were not living with a partner. More than half (54.0%) had completed secondary education, and 70.5% reported menstruating within the previous 12 months. Regarding work shifts, the majority (86.8%) were day-shift workers. In terms of behavioral factors, 70.5% reported not engaging in physical activity, and 47.0% rated their health as good. Concerning dietary habits, 61.9% of the participants reported consuming four or more meals per day. As for medication use, 90.4% did not use sleep aids, and 95.7% did not take medication for diabetes mellitus. With respect to anthropometric indicators, most participants were classified with overweight (37.4%) or with obesity (30.1%), and 46.6% presented abdominal obesity.

As shown in Table 1, female night-shift workers exhibited significantly higher mean serum leptin levels (48.8 ± 48.6 ng/mL; *p* < 0.001) compared with day-shift workers. Regarding physical activity, participants who did not engage in regular exercise had higher mean leptin concentrations than those who reported being physically active (36.3 ± 28.8 ng/mL; *p* = 0.006). In terms of self-rated health, women who classified their health as fair or poor demonstrated higher mean serum leptin levels (40.3 ± 33.8 ng/mL; *p* = 0.02). With respect to dietary habits, participants who reported consuming three or fewer meals per day presented higher mean leptin concentrations (39.6 ± 34.5 ng/mL; *p* = 0.02) compared to those consuming four or more meals daily. Women with obesity also showed markedly elevated mean serum leptin levels (55.1 ± 34.8 ng/mL; *p* < 0.001), as did those with abdominal obesity (45.2 ± 30.3 ng/mL; *p* < 0.001).

The mean serum leptin concentration in the sample was 33.6 ng/mL (95% CI: 30.6–36.6), and the prevalence of altered serum leptin levels was 78.1% (95% CI: 73.5–82.8). The results of the multivariate analysis are summarized in Table 2. After adjustment for potential confounders, women with obesity had a 63% higher probability of presenting altered serum leptin levels (PR = 1.63; 95% CI: 1.32–2.02; *p* < 0.001) compared with those without obesity. Although women with abdominal obesity exhibited a higher mean serum leptin concentration (45.2 ng/mL) than those without abdominal obesity (23.8 ng/mL; *p* < 0.001), this association was not statistically significant after multivariate adjustment (PR = 1.10; 95% CI: 0.97–1.24; *p* = 0.14). Additionally, night-shift workers exhibited a 14% higher occurrence of altered serum leptin levels compared with day-shift workers (PR = 1.14; 95% CI: 1.01–1.30; *p* = 0.05). Conversely, engaging in regular physical activity was associated with a 20% lower occurrence of altered leptin levels (PR = 0.80; 95% CI: 0.68–0.94; *p* = 0.01) (Table 2).

**Figure 1:**
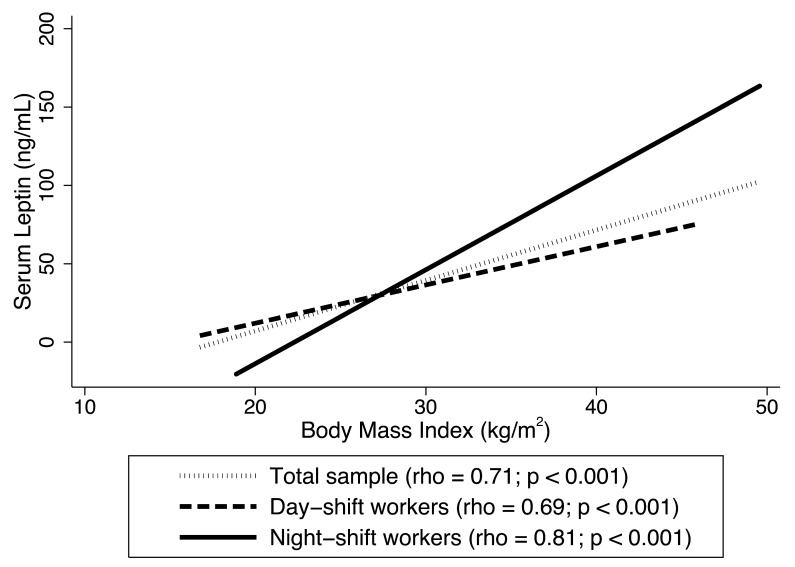
Spearman’s rank correlation coefficient (rho) between leptin levels and body mass index (BMI) among Brazilian female shift workers, 2022 (*n* = 302).

Figure 1 presents the correlation between leptin levels and BMI for the total sample and stratified by work shift. A positive and statistically significant correlation was observed in the total sample (rho = 0.71; *p* < 0.001). A similar correlation was found among day-shift workers (rho = 0.69; *p* < 0.001), while a higher stronger correlation was observed among night-shift workers (rho = 0.81; *p* < 0.001).

## 4. Discussion

In this study, we revealed a high prevalence of altered serum leptin levels (>15.2 ng/mL) among female shift workers employed in an industrial group located in southern Brazil, with a mean concentration of 33.6 ng/mL and 78.1% of participants with altered serum leptin levels. After multivariate adjustment, women with obesity showed a 63% higher probability of presenting altered leptin levels compared with those without obesity.

The increase in adipose tissue associated with obesity is accompanied by elevated leptin concentrations [31]. As leptin is synthesized primarily by adipocytes, higher circulating levels of this hormone reflect greater fat accumulation [32]. Individuals with obesity often exhibit elevated free leptin levels in the brain, which may contribute to the development of leptin resistance—a condition characterized by reduced sensitivity or impaired responsiveness to leptin signaling. This resistance diminishes leptin’s anorexigenic effects, promoting increased food intake and further weight gain [33]. The present findings reinforce this physiological relationship, underscoring the strong association between adiposity and elevated serum leptin concentrations.

Previous research has reported a potential relationship between central adiposity and leptin concentration [34,35]. For instance, a study involving 141 Korean women found higher leptin levels among individuals with obesity and a positive correlation between leptin and waist circumference [34]. Similarly, another investigation including 158 participants examined metabolic hormones and adipokines in relation to abdominal obesity, revealing that alterations in these biomarkers are also linked to central fat accumulation [35]. In the present study, the prevalence of abdominal obesity was 46.6%. Women with abdominal obesity had a mean serum leptin concentration of 45.2 ng/mL, compared with 23.8 ng/mL among those without abdominal obesity. However, after statistical adjustment, no significant association was observed between abdominal obesity and altered serum leptin levels. 

The findings of this study align with previous evidence showing that night-shift work is associated with metabolic alterations. In the present analysis, night-shift workers exhibited a 14% higher prevalence of altered leptin levels compared with day-shift workers, suggesting an increased risk for metabolic disturbances. Similarly, a cross-sectional study conducted in Karnataka, India, with 88 industrial workers found that night-shift work was significantly associated with elevated leptin levels and a higher prevalence of metabolic syndrome [24].

Night-shift work has been consistently associated with adverse health outcomes, primarily due to circadian misalignment [20,21]. This disruption leads to fatigue, reduced alertness, and increased risk of metabolic and cardiovascular disorders [20,21]. Sleep disturbances related to shift work alter hormonal regulation and promote a positive energy balance. Sleep restriction may induce metabolic and endocrine changes, including decreased insulin sensitivity, elevated nocturnal cortisol, increased ghrelin, and reduced leptin levels, collectively enhancing hunger and appetite [21]. In southern Brazil, a previous cross-sectional study with 450 female shift workers reported a prevalence of abdominal obesity of 56.1% among night-shift workers compared with 40.9% among hybrid-shift workers, emphasizing the need for behavioral strategies such as increased meal frequency and regular physical activity [36]. 

Physical activity has been shown to reduce inflammation and improve metabolic regulation associated with adiposity. In a U.S. cohort of nearly 2000 adults, moderate to vigorous physical activity was linked to approximately 30% lower leptin levels, reflecting a more favorable inflammatory profile [37]. Experimental studies further suggest that exercise activates leptin receptor–positive neurons in the ventromedial hypothalamus, enhancing leptin sensitivity and energy balance independent of fat mass [38]. A meta-analysis of randomized clinical trials confirmed that physical activity, alone or combined with dietary changes, significantly decreases circulating leptin concentrations [39]. Similarly, cross-sectional data from 536 women in the U.S. indicated that higher activity levels were associated with lower leptin and insulin concentrations and reduced cardiovascular risk [40]. These findings are consistent with the present study, in which engaging in physical activity was associated with a 20% lower prevalence of altered leptin levels.

In our study, we examined a broad set of potential covariates—including sociodemographic, behavioral, reproductive, and health-related characteristics—to better contextualize the variability in circulating leptin levels. Although several of these factors did not show statistically significant associations in our sample, prior evidence highlights important patterns that warrant consideration. For example, leptin concentrations tend to decline with advancing age, independent of BMI, with this decrease being more pronounced in women [41]. Racial differences have also been reported, with Black women exhibiting higher leptin levels than White women after adjustment for adiposity and other confounders [42]. In contrast, marital status has not been linked to leptin variation, with one previous study reporting no significant differences between married and unmarried women [43]. Regarding reproductive factors, observational studies have documented inconsistent patterns in leptin fluctuations across the menstrual cycle, suggesting substantial interindividual variability [44]. Moreover, previous research has identified an association between leptin and self-rated health, with higher leptin levels being more strongly linked to better perceived health among women compared with men [45]. Finally, experimental evidence suggests that eating frequency may modulate leptin rhythmicity independently of total caloric intake [46]. Taken together, these findings underscore the complex, multifactorial regulation of leptin and reinforce the importance of considering a wide range of biopsychosocial variables when interpreting leptin–adiposity relationships in specific populations, such as female shift workers.

**Figure 2:**
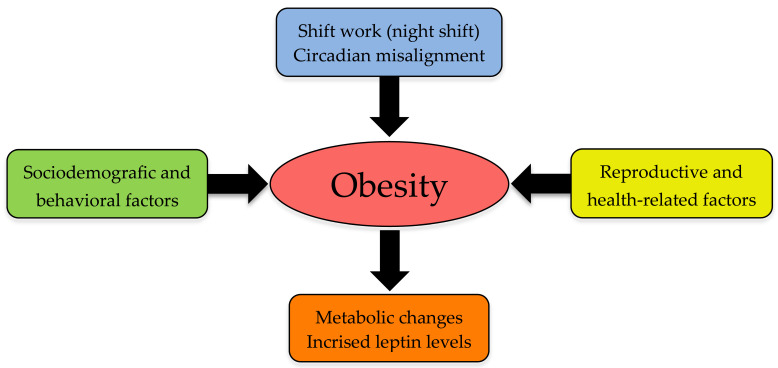
Schematic disease-model pathways linking obesity, metabolic alterations, and leptin levels in shift workers.

Figure 2 presents the disease-model pathways proposed in this study, outlining the interrelations among obesity, metabolic alterations, and leptin levels in shift workers.

This study presents several noteworthy strengths. To our knowledge, this is one of the first studies to examine the association between obesity and leptin in a specific sample of women exposed to shift work. By focusing on this occupational group, the study contributes to the existing literature by addressing a population that may be particularly vulnerable to hormonal and metabolic disturbances. Our findings advance current knowledge on the potential mechanisms linking shift work, adiposity, and alterations in leptin regulation, thereby enhancing the understanding of metabolic health risks among female shift workers. The use of standardized data collection protocols, including anthropometric and biochemical assessments performed by trained professionals, ensured methodological rigor, and minimized measurement bias. The laboratory-based assessment of leptin levels, combined with the inclusion of behavioral, reproductive, and health-related variables, enabled a comprehensive evaluation of potential confounding factors. Furthermore, the application of a hierarchical analytical model strengthened the interpretation of associations across multiple levels of influence. Collectively, these features enhance the study’s reliability and internal validity. Nonetheless, certain limitations should be acknowledged. This was a cross-sectional study involving the simultaneous measurement of exposure and outcome, which limits causal inferences and does not allow the exclusion of potential reverse causality. The study sample was restricted to female employees from a single industrial company located in southern Brazil, highlighting the low generalizability of the findings to other occupational contexts or to male populations, for example. Moreover, the exclusive inclusion of fixed-shift workers precluded the examination of potential effects associated with rotating schedules. A further limitation is that obesity classification was based on BMI derived from duplicate height and weight measurements; thus, BMI-based categorization may not fully correspond to a physician-confirmed clinical diagnosis, potentially introducing some degree of misclassification. This study also did not assess potential confounding factors such as emotional stress, elevated glucocorticoid levels, menstrual cycle phase, or the use of specific sleep medications for conditions such as obstructive sleep apnea, which may influence circulating leptin concentrations. Finally, leptin concentrations were measured at a single time point that varied across the workday according to participants’ shifts, without accounting for intraday circadian variation—a factor that may influence serum leptin levels and affect the interpretation of single–time–point measurements. Thus, we recommend that these aspects be carefully considered in future research, with particular emphasis on the need for studies involving a larger number of companies and incorporating longitudinal analyses to better elucidate the relationship between obesity and elevated leptin levels in this population.

## 5. Conclusions

Obesity was independently associated with elevated serum leptin levels among female shift workers, suggesting that excess adiposity remains a key determinant of leptin dysregulation in this population. Although abdominal obesity did not retain statistical significance after adjustment, lifestyle, and occupational factors—particularly shift work and physical activity—appeared to modulate leptin concentrations. These findings underscore the vulnerability of women engaged in shift work—particularly those at greater metabolic risk, such as workers with obesity, those assigned to night shifts, and those who do not engage in regular physical activity—and highlight the need for workplace health initiatives tailored to mitigate these risks. Such initiatives may include organizational policies that promote more consistent meal timing, controlled-lighting environments to support circadian adaptation, and opportunities for physical activity during or between shifts. Integrating these measures into occupational health programs may help reduce metabolic risk and improve long-term health outcomes among female shift workers. Moreover, as work is a social determinant of health, discussions on adequate remuneration, working-hour arrangements, and shift-work characteristics remain essential.

## Figures and Tables

**Table 1:** Sample characteristics, mean and standard deviation of serum leptin, and prevalence of altered serum leptin (>15.2 ng/mL) according to sample characteristics among Brazilian female shift workers, 2022 (*n* = 302).

Characteristics		Serum Leptin (ng/mL)			Prevalence of Altered Leptin	
	*n* (%)	Mean	SD	*p*-Value *	%	*p*-Value **
Age				0.83		0.67
18–30 years	106 (35.1)	34.3	26.6		78.3	
31–40 years	103 (34.1)	34.8	26.4		80.6	
≥41 years	93 (30.8)	32.2	26.7		75.3	
Skin color				0.30		0.74
White	210 (69.5)	34.6	28.3		77.6	
Other	92 (30.5)	31.2	21.7		79.3	
Marital status				0.82		0.64
Without partner	157 (52)	33.9	26.7		77.1	
With partner	145 (48)	33.2	26.3		79.3	
Educational level				0.15		0.63
Primary school	21 (7.0)	39.6	29.7		85.7	
Secondary school	163 (54)	31.0	23.4		76.7	
Technical/higher	118 (39.1)	36.1	29.5		78.8	
Menstruated in the last 12 months				0.45		0.16
Yes	213 (70.5)	34.3	25.5		80.3	
No	89 (29.5)	31.8	28.7		73.0	
Work shift				<0.001		0.01
Day shift	262 (86.8)	31.2	20.3		76.7	
Night shift	40 (13.2)	48.8	48.6		87.5	
Physical activity				0.006		0.001
No	213 (70.5)	36.3	28.8		83.1	
Yes	89 (29.5)	27.1	18.2		63.3	
Self-perception of health				0.02		0.14
Excellent/Very good	73 (24.2)	30.2	19.1		78.1	
Good	142 (47)	31.2	23.9		73.4	
Fair/poor	87 (28.8)	40.3	33.8		85.1	
Number of meals per day				0.02		0.54
3 or fewer	115 (38.1)	39.6	34.5		80.0	
4 or more	187 (61.9)	29.8	19.2		77.0	
Use of sleeping medication				0.15		0.75
Yes	29 (9.6)	40.3	32.0		75.9	
No	273 (90.4)	32.9	25.7		78.4	
Use of medication for diabetes				0.30		0.20
Yes	13 (4.3)	41.0	15.0		92.3	
No	289 (95.7)	33.3	26.8		77.5	
BMI classification				<0.001		<0.001
Normal	98 (32.5)	17.6	10.9		53.1	
Overweight	113 (37.4)	30.1	13.8		85.0	
Obesity	91 (30.1)	55.1	34.8		96.7	
Abdominal obesity				<0.001		<0.001
No	158 (53.4)	23.8	17.6		64.6	
Yes	138 (46.6)	45.2	30.3		94.3	

SD, Standard Deviation; BMI, Body Mass Index; * *t* test or ANOVA test for comparison of means; ** Pearson’s Chi-square test for heterogeneity of proportions.

**Table 2:** Multivariate analysis for the association between general and abdominal obesity and altered serum leptin (>15.2 ng/mL) among Brazilian female shift workers, 2022 (*n* = 302).

	Altered Serum Leptin (>15.2 ng/mL)	
Characteristics	PR (95% CI)	*p*-Value
**Level 1—Demographic, socioeconomic, and reproductive variables**		
Education		0.65
Primary school	1.00	
Secondary school	0.89 (0.73–1.07)	
Technical/higher	0.89 (0.74–1.09)	
Menstruated in the last 12 months		0.18
No	1.00	
Yes	1.10 (0.95–1.27)	
**Level 2—Work shift**		
Work shift		0.05
Day shift	1.00	
Night shift	1.14 (1.01–1.30)	
**Level 3—Behavioral variables**		
Physical activity		0.01
No	1.00	
Yes	0.80 (0.68–0.94)	
Self-perceived health		0.67
Excellent/very good	1.00	
Good	0.90 (0.77–1.06)	
Fair/poor	1.01 (0.87–1.18)	
Number of meals per day		0.99
3 or fewer	1.00	
4 or more	1.01 (0.89–1.13)	
**Level 4—Health variables**		
Use of sleeping medication		0.33
Yes	1.00	
No	0.92 (0.74–1.13)	
Use of medication for diabetes		0.40
No	1.00	
Yes	1.08 (0.93–1.26)	
BMI classification		<0.001
Normal	1.00	
Overweight	1.55 (1.27–1.91)	
Obesity	1.63 (1.32–2.02)	
Abdominal obesity		0.14
No	1.00	
Yes	1.10 (0.97–1.24)	

PR, Prevalence Ratios with 95% Confidence Intervals (95% CI) obtained by Poisson regression with robust variance. Level 1: sociodemographic and reproductive variables: education level and menstruation in the last 12 months. Level 2: variables from level 1 with *p*-value < 0.20 (menstruation in the last 12 months) + work shift. Level 3: variables from level 2 with *p*-value < 0.20 (menstruation in the last 12 months and work shift) + physical activity, number of meals, and self-perceived health. Level 4: variables from level 3 with *p*-value < 0.20 (menstruation in the last 12 months, work shift, and physical activity) + use of sleeping medication, use of medication for Diabetes Mellitus, BMI classification, and abdominal obesity.

## Data Availability

The data presented in this study are available upon reasonable request to the corresponding author.

## Abbreviations

The following abbreviations are used in this manuscript:

BMI	Body Mass Index
WC	Waist Circumference
CI	Confidence Intervals
PR	Prevalence Ratios
EIA	Enzyme Immunoassay
